# Macro-chéilite granulomatose révélant une maladie de Crohn

**DOI:** 10.11604/pamj.2018.30.147.5395

**Published:** 2018-06-20

**Authors:** Khaoula Jellali, Ihsane Mellouki, Adil Ibrahimi

**Affiliations:** 1 Service d’Hépato-gastroentérologie, Faculté de Médecine et de Pharmacie de Fès, Université Sidi Mohammed Ben Abdellah, Fès, Maroc

**Keywords:** Maladie de chron, cheilite granulomateuse, diagnostic, Crohn’s disease, cheilitis granulomatosa, diagnosis

## Abstract

De nombreuses affections du tube digestif comportent des manifestations cutanées qui peuvent être révélatrices, ce qui place le dermatologue en première ligne pour orienter le patient vers la consultation de gastroentérologie. A l’inverse, le gastroentérologue est souvent confronté à des lésions cutanéo-muqueuses pour lesquelles se pose la question d'une éventuelle relation avec une maladie digestive déjà connue. Les maladies inflammatoires chroniques de l'intestin représentent l’exemple type de cette nécessaire collaboration entre les deux spécialités. Nous rapportons un cas de maladie de crohn révélée par une Chéilite granulomateuse. Il s'agit d'une patiente âgée de 30 ans. Suivie en dermatologie pour une chéilite granulomateuse. L’examen somatique était sans particularité mise à part les lésions dermatologiques (un œdème facio-labiale blanc, induré et une gingivite diffuse). La biopsie de la lésion labiale a objectivé des granulomes épithélioides et gigantocellulaire à disposition péri-vasculaire sans nécrose caséeuse. Devant la suspicion d’une colite inflammatoire une iléo-coloscopie a été réalisée ayant objectivé une valvule de Bauhin béante et ulcérée avec une muqueuse iléale légèrement érythémateuse, dont l’étude anatomopathologique avait révélé un aspect histologique en faveur d'une maladie inflammatoire chronique de l’intestin type crohn iléo-colique. L’évolution était marquée par l'installation, 6 mois après, de quelques épisodes de réctorragies avec lésions ano-périnéales: large fissure anale antérieure avec une ulcération aphtoide à 5 cm de la marge anale, compliquée quelques semaines après par l'installation d'un abcès péri-anale fistulisé ayant nécessité un drainage chirurgicale avec mise en place de Sétons, d’où l’indication de démarrer un traitement de fond de sa maladie de crohn après assèchement de l’abcès. Les lésions dermatologiques observées lors des MICI sont très variées. Dans certains cas, elles apparaissent au cours d'une MICI connue alors que, dans d'autres, elles précédent ou accompagnent les manifestations digestive, permettant le diagnostic d'une affection intestinale parfois cliniquement latente.

## Abstract (en)

Many disorders of the digestive tract cause cutaneous manifestations that may be an indication of an underlying condition; hence dermatologist plays a key role in recommending that the patient see a gastroenterologist. Conversely, gastroenterologist often sees patients with mucocutaneous lesions suggesting possible association with well-known digestive disorders. Chronic inflammatory bowel diseases are the typical example of this essential collaboration between the two specialities. We report a case of Crohn’s disease revealed by cheilitis granulomatosa. This study involved a 30-year old female patient followed up for cheilitis granulomatosa in the Department of Dermatology. Somatic examination was unremarkable apart from dermatologic lesions (indurated white faciolabial edema and diffuse gingivitis). Biopsy of the labial lesion revealed perivascular epithelioid and giant-cell granulomas without caseous necrosis. Ileocolonoscopy was performed due to suspected inflammatory colitis. It showed gaping and ulcerated Bauhin valve with slightly erythematous ileal mucosa. Anatomo-pathologic study objectified chronic inflammatory bowel disease (CIBD) such as ileocolic Crohn’s disease. Patient’s evolution was marked by some episodes of rectal bleeding with anoperineal lesions 6 months later: a wide anterior anal fissure with aphthous ulceration 5 cm from the anal margin, complicated, a few weeks later, by the onset of perianal fistulized abscess requiring surgical drainage and seton installation. Hence the indication for thorough treatment of Crohn’s disease after complete drying up of the abscess. Dermatologic lesions vary in patients with CIBD. In some cases, they occur in patients with IBD whereas, in other cases, they precede or accompany digestive manifestations, allowing diagnosis of sometimes clinically latent bowel disease.

## Introduction

De nombreuses affections du tube digestif comportent des manifestations cutanées qui peuvent être révélatrices, ce qui place le dermatologue en première ligne pour orienter le patient vers la consultation de gastroentérologie. A l'inverse, le gastroentérologue est souvent confronté à des lésions cutanéo-muqueuses pour lesquelles se pose la question d'une éventuelle relation avec une maladie digestive déjà connue. Les maladies inflammatoires chroniques de l'intestin représentent l'exemple type de cette nécessaire collaboration entre les deux spécialités [1]. Les manifestations cutanéo-muqueuses et osteo-articulaires sont les plus fréquentes des atteintes extradigestives des MICI. Les lésions dermatologiques observées sont très variées. Dans certains cas, elles apparaissent au cours d'une MICI connue alors que, dans d'autres, elles précédent ou accompagnent les manifestations digestive, permettant le diagnostic d'une affection intestinale parfois cliniquement latente [2]. La fréquence des manifestations cutanéo-muqueuses est très variable selon les séries, avec des extrêmes tres éloignés: de 2 à 85 % [3]. Les lésions oro-faciales peuvent se présenter selon plusieurs aspect: des ulcérations linéaires a bords hyperplasiques des sillons gingivo-jugaux, des ulcérations de présentations trompeuses car prenant l'aspect d'aphtes, des lésions polypoides de la muqueuse vestibulaire ou jugale, une hyperplasie œdémateuse et fissurée de la face interne des joues, des lèvres, réalisant un aspect en «pavée», une chéilite granulomateuse qui se manifeste par un œdème induré d'une ou des deux lèvres, épisodique au début puis permanant. L'atteinte labiale est habituellement asymétrique fissuraire et s'accompagne d'une perlèche. Les biopsies profondes avec réalisation de nombreux plans de coupes sont nécessaires pour mètre en évidence des petits granulomes spécifiques. En l'absence d'arguments pour une sarcoïdose, il faut réaliser un bilan digestif au moindre signe d'appel car cette chéilite granulomateuse est souvent précoce et peut précéder de plusieurs années les manifestations intestinales [1].

## Patient et observation

**Figure 1:**
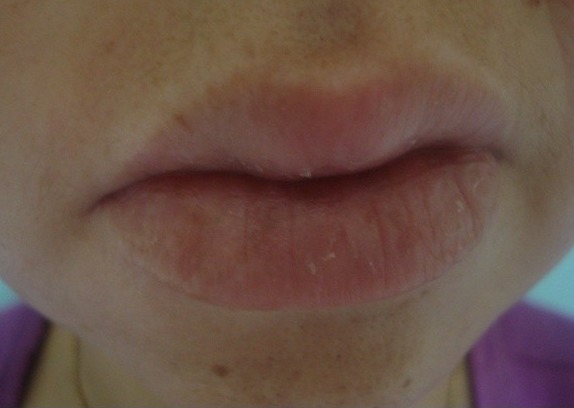
œdème labial homogène et symétrique

**Figure 2:**
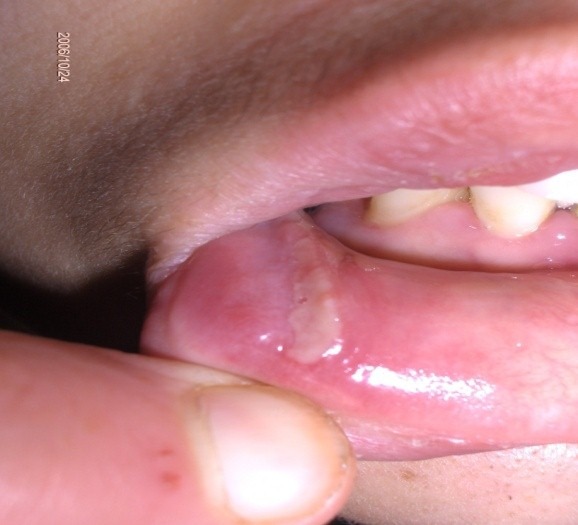
Une gingivite diffuse, une lésion aphtoïde buccale

**Figure 3:**
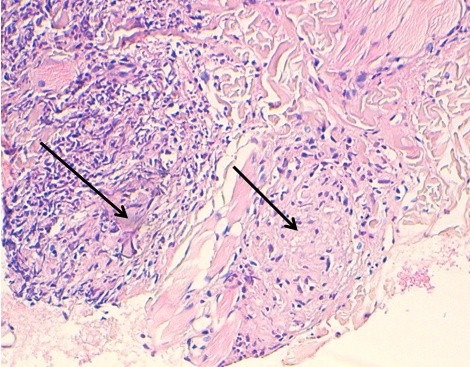
Granulomes épithéloides et giganto-cellulaire à disposition péri vasculaire sans nécrose caséeuse

Il s'agit d'une jeune patiente de 30 ans, adressée en dermatologie pour un œdème labial récurrent depuis 2 ans. La patiente était asymptomatique sur le plan digestif. L'examen clinique avait objectivé un œdème labial homogène et symétrique (Figure 1); une gingivite diffuse, une lésion aphtoide buccale (Figure 2), un placard eczématiforme rétro-auriculaire et une xérose. Par ailleurs; on ne trouvait ni macroglossie ni langue plicaturée ni paralysie faciale. Les biopsies de la macrochéilite et de la lésion aphtoïde étaient réalisées (Figure 3) et dont l'étude anatomopathologique avait objectivé des granulomes épithélioides et giganto-cellulaire à disposition péri-vasculaire sans nécrose caséeuse. Dans le cadre du bilan étiologique et devant la présence de diarrhées chronique intermittentes nous avons complété par une Iléocoloscopie qui a objectivé une valvule de Bauhin (iléo-caecale) béante ulcérée; une muqueuse iléale légèrement érythémateuse avec une muqueuse colique d'aspect macroscopiquement normal. Les biopsies ileales et coliques étagées avaient objectivé des anomalies épithéliales iléo-colique à type de décollement avec granulome épithélioide et gigantocellulaire sans nécrose caséeuse. De ce fait, le diagnostic de la maladie de crohn latente a été retenu. La patiente était suivie régulièrement avec abstinence thérapeutique étant donné que la pathologie digestive était en période de rémission clinique. Concernant la macrocheilite; une abstention thérapeutique a été indiqué également car elle était homogène et psychologiquement tolérable par la patiente. L'évolution était marquée par l'installation 6 mois après d'un abcès anale avec une diarrhée chronique; ce qui a motivé l'indication d'un traitement immunosuppresseur à base d'Azathioprine après cure chirurgicale de l'abcès. On notait par ailleurs une amélioration progressive de la macrocheilite après un recul de 6 mois.

## Discussion

La macrochéilite granulomateuse est une inflammation chronique des lèvres caractérisée par un œdème induré d'une ou deux lèvres, épisodique au début puis permanant. L'atteinte labiale est habituellement asymétrique, fissuraire, et s'accompagne de perlèche. Les biopsies profondes avec réalisation de nombreux plans de coupe sont nécessaire pour mettre en évidence les granulomes épithélioïde et giganto-cellulaire sans nécrose caséeuse [1,4]. La maladie de crohn peut se manifester par une macrocheilite granulomateuse pouvant précéder de plusieurs années l'atteinte intestinale. Un traitement «préventif » des troubles digestifs ne se justifie pas en raison du rapport bénéfice /risque du traitement immunosuppresseur. Les manifestations dermatologiques sont retrouvées chez 22 à 44% des patients, et sont les atteintes extradigestives les plus fréquentes au cours des maladies inflammatoires du tube digestif [1,2]. Le traitement de la macrocheilite granulomateuse est non codifié et dépend de la demande du patient. Les traitements symptomatiques ont pour but d'améliorer la qualité de vie du malade, mais le traitement reste étiologique [2,5].

## Conclusion

Les lésions dermatologiques observées lors des MICI sont très variées. Dans certains cas, elles apparaissent au cours d'une MICI connue alors que, dans d'autres, elles précédent ou accompagnent les manifestations digestive, permettant le diagnostic d'une affection intestinale parfois cliniquement latente.

## Conflits d’intérêts

Les auteurs ne déclarent aucun conflit d'intérêts.
